# Proton Pump Inhibitor Pantoprazole Modulates Intestinal Microbiota and Induces TLR4 Signaling and Fibrosis in Mouse Liver

**DOI:** 10.3390/ijms232213766

**Published:** 2022-11-09

**Authors:** Heloisa B. Assalin, Kelly Cristiane Gabriel De Almeida, Dioze Guadagnini, Andrey Santos, Caio J. Teixeira, Silvana Bordin, Guilherme Z. Rocha, Mario J. A. Saad

**Affiliations:** 1 Department of Internal Medicine, School of Medical Sciences, University of Campinas, Campinas 13080-655, SP, Brazil; 2 Department of Physiology and Biophysics, Institute of Biomedical Science, University of Sao Paulo, Sao Paulo 05508-000, SP, Brazil

**Keywords:** microbiota, proton pump inhibitors, liver steatosis

## Abstract

Proton pump inhibitors (PPIs) are one of the most prescribed drugs around the world. PPIs induce microbiota modulation such as obesity both in humans and in animal models. However, since PPIs can induce microbiota modulation despite the absence of a high-fat diet or weight gain, it is an interesting model to correlate microbiota modulation with the establishment of non-alcoholic fatty liver disease (NAFLD). We investigated the effect of pantoprazole treatment on TLR4 signaling and liver histology in C57BL/6J mice for 60 days, trying to correlate microbiota modulation with some aspects of liver injury. We performed glucose (GTT) and insulin (ITT) tolerance tests, serum lipopolysaccharide (LPS) dosage, liver histology, liver and intestine extraction for Western blot and qPCR. Fecal microbiota were investigated via metagenomics. Chronic treatment with pantoprazole induced microbiota modulation and impaired ileum barrier integrity, without an association with insulin resistance. Furthermore, increased circulating LPS and increased Toll-like receptor 4 (TLR4) and TGFβ downstream signaling may have an important role in the development of the observed liver microvesicular steatosis and fibrosis. Finally, this model of PPI-induced changes in microbiota might be useful to investigate liver microvesicular steatosis and fibrosis.

## 1. Introduction

PPIs are one of the most prescribed drugs around the world [1,2,3,4] to treat heartburn, gastroesophageal reflux disorder (GERD) and to prevent gastroduodenal ulcers [2,3,4]. Most of the time, these drugs are prescribed for long periods without weighing up the risks and benefits. The list of adverse events induced by these drugs has increased in the past few years, but it is important to mention that only a few have established causalities [5,6,7,8,9]. 

PPIs have clear effects on structural and functional changes in the gastric mucosa, but the relationship between these drugs and gastrointestinal malignancies is not evidence-based [10,11,12,13]. On the other hand, previous data showed that chronic PPI use could be accompanied by an increased risk of enteric infections [9]. These data are supported by epidemiologic studies and meta-analyses, and the mechanism seems to be related to changes induced by these drugs on the gut microbiota [14,15,16,17,18,19,20,21,22]. The microbiota modulation induced by PPIs is very similar to microbiota seen in animal models of obesity and human obesity [23,24,25,26,27]. In this regard, changes in microbiota composition have been causally related to obesity and its complications [23,24,25,26]. 

NAFLD is one of the most common complications of obesity, and its prevalence (from 1989 to 2017) in the world population has ranged from 11.2% to 37.2% [28,29,30]. In the past five years, data coming from different sources have shown that dysbiosis has an important role in the development of NAFLD [28,29,30]. The mechanisms by which microbiota modulation can have a role in the induction of NAFLD include the reduced production of short-chain fatty acids produced by bacteria and increased serum LPS, indicating an alteration in the intestinal barrier, which will induce liver inflammation through TLR4 [23]. NAFLD can progress to NASH, which is characterized by advanced fibrosis, and this process seems to be influenced by the microbiota [28,29,30,31,32]. Recent evidence suggests a link between NAFLD and PPI use, probably through gastric achlorhydria which induces alterations in microbiota, but these studies were performed in alcohol-fed mice and in obese mice [33,34].

It is important to mention that most animal models of NAFLD and NASH use a high-fat diet or high-caloric diet, which by themselves can modulate microbiota, making it difficult to establish a direct correlation between gut microbiota with NAFLD. However, since PPIs can induce microbiota modulation similar to obesity, but without a high-fat diet or weight gain, we believe that it is an interesting model to correlate microbiota modulation with liver injury. Thus, we investigated the effect of pantoprazole treatment for 60 days on glucose metabolism, the intestinal barrier integrity, associated with TLR4 signaling, and liver histology, trying to correlate microbiota modulation with some aspects of liver injury.

## 2. Results

### 2.1. Animal Characterization

**Figure 1:**
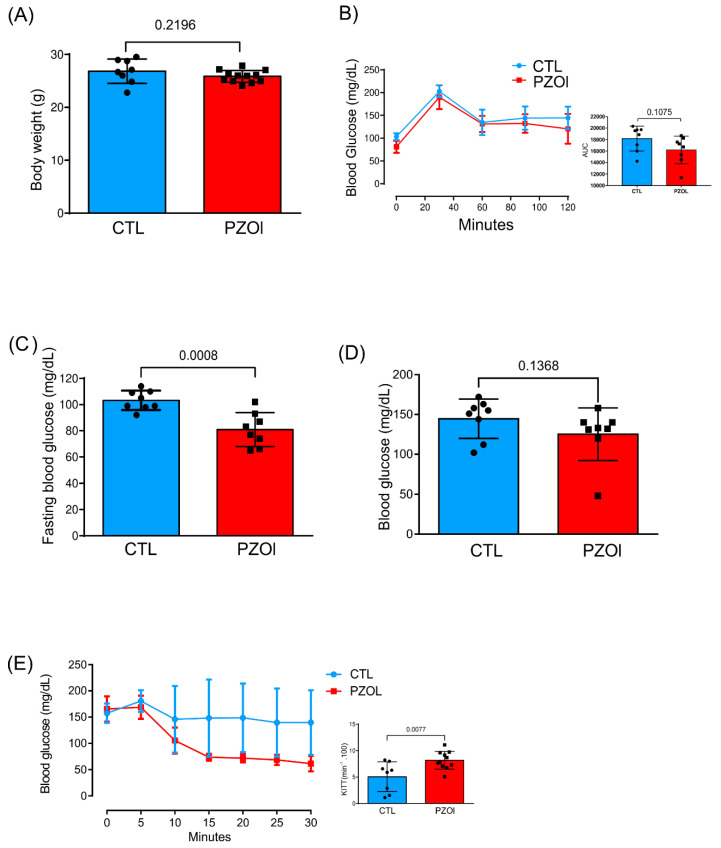
Effect of pantoprazole treatment. (**A**) Body weight (CTL: *n* = 8 and PZOL: *n* = 12); (**B**) GTT curve and AUC (CTL: *n* = 8 and PZOL: *n* = 8); (**C**) fasting blood glucose (CTL: *n* = 8 and PZOL: *n* = 8); (**D**) blood glucose 120 min after glucose injection (CTL: *n* = 8 and PZOL: *n* = 8); (**E**) kITT, from CTL and PZOL-treated mice (CTL: *n* = 8 and PZOL: *n* = 11). Data are expressed as mean ± standard deviation and *t*-test statistical comparisons.

Pantoprazole-treated (PZOL) mice presented similar body weights at the end of treatment when compared to vehicle-treated mice (CTL) (CTL: 26.84 ± 2.289 and PZOL: 25.86 ± 1.136; *p*-value 0.2196; Figure 1A). Furthermore, the GTT also presented similar curves (Figure 1B) without any difference in the area under the curve (CTL: 18,174 ± 2171 and PZOL: 16,202 ± 2411; *p* = 0.1075). On the other hand, the fasting blood glucose was lower in PZOL mice than in CTL mice (CTL: 103.3 ± 7.44 and PZOL: 80.88 ± 12.93; *p*-value 0.0008; Figure 1C), and it also showed a tendency to be lower at the end of the GTT, i.e., 120 min after glucose infusion (CTL: 153 ± 24.8 and PZOL: 132 ± 33.0; *p*-value 0.1368; Figure 1D). In addition, pantoprazole treatment was able to improve insulin sensitivity, as shown by the increase in kITT (CTL: 5.057 ± 2.820 and PZOL: 8.171 ± 1.674; *p*-value 0.0077: Figure 1E).

### 2.2. Microbiome Data Analysis after 60 Days of Pantoprazole

**Figure 2:**
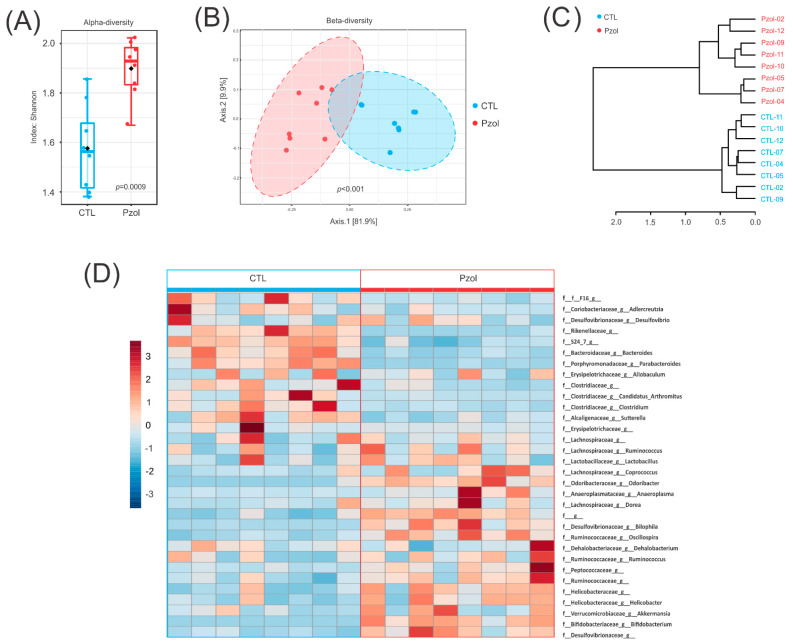
Community profiling and clustering analysis. (**A**) Alpha diversity measure using Shannon diversity index at genus level represented as boxplot. Each boxplot represents the diversity distribution of a group (*p*-value: 0.0009—Mann–Whitney); (**B**) beta diversity. Principal coordinate analysis (PCoA): comparison of between-community diversity based on Bray–Curtis distance parameter. *p*-value for each comparison was obtained from PERMANOVA and considered significant at *p*-value  <  0.05. [PERMANOVA] F-value: 31.518; R-squared: 0.69243; *p*-value < 0.001; (**C**,**D**) hierarchical cluster analysis: each sample begins as a separate cluster and the algorithm proceeds to combine them until all samples belong to one cluster; (**C**) dendrogram; and (**D**) heatmap. (*n* = 8 animals per group).

To investigate how pantoprazole treatment, for 60 days, affected microbiota, first, we analyzed the α-diversity, estimated by the Shannon index. This method is used to measure the diversity present within a sample or community. The Shannon index considered two measures: richness (total number of species) and evenness (abundances of the species). The alpha diversity was higher in the PZOL group when compared with the CTL group (*p*-value 0.0009; Figure 2A). 

Then, we analyzed the beta diversity, which provides a way to compare the diversity or composition between two samples or microbial communities. This analysis was performed using the phyloseq package [35]. Principal coordinate analysis (PCoA) based on the Bray–Curtis distance parameter showed the essential difference between the PZOL group and the CTL group. A *p*-value for each comparison was obtained from PERMANOVA and considered significant at *p*-value < 0.05. (PERMANOVA F-value: 31.518; R-squared: 0.69243; *p*-value < 0.001; Figure 2B). 

In hierarchical cluster analysis, each sample begins as a separate cluster and the algorithm proceeds to combine them until all samples belong to one cluster. The hierarchical cluster analysis at the genus level was based on Bray–Curtis metrics and Ward’s linkage (clustering to minimize the sum of squares of any two clusters). The sample hierarchical cluster analysis (dendrogram and heatmap) showed that the PZOL group and CTL group belonged to two clusters (Figure 2C,D). 

**Figure 3:**
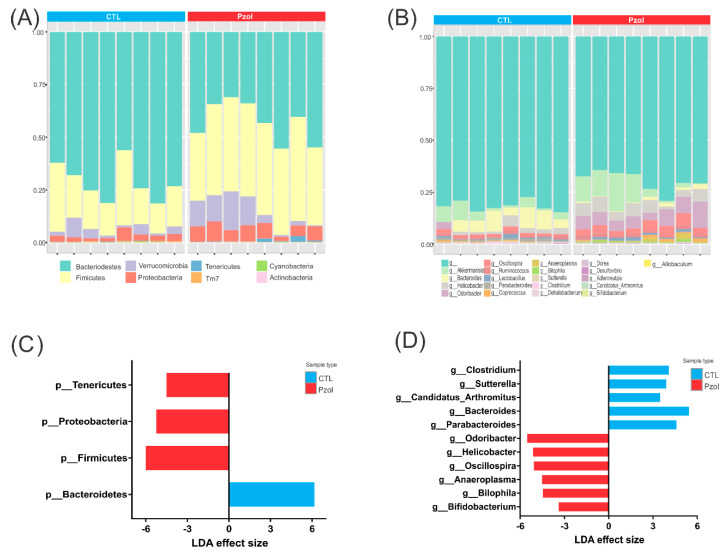
Taxonomic composition of community and LEfSe at phyla or genus level. Taxonomic composition of community through direct quantitative comparison of abundances of phyla (**A**) and genus (**B**) using stacked bar plot at individual sample level. LDA Effect Size (LEfSe) for phyla (**C**) and genus (**D**). LDA was used for biomarker discovery and explanation of high-dimensional metagenomic data. It incorporates statistical significance with biological consistency (effect size) estimation. The default was adjusted *p*-value cutoff = 0.05. (*n* = 8 animals per group).

The taxonomic composition at the phylum and genus level can be viewed at the individual-sample level (Figure 3A,B). LDA Effect Size (LEfSe) was used for the biomarker discovery and explanation of high-dimensional metagenomic data. It incorporated statistical significance with biological consistency (effect size) estimation. It performed a non-parametric factorial Kruskal–Wallis (KW) sum-rank test, and the default was an adjusted *p*-value cutoff = 0.05. The LEfSe analysis confirmed the differences between the PZOL and CTL group and identified a total of three phyla enriched in the PZOL group (Firmicutes, Tenericutes and Proteobacteria) and just one phylum in the CTL group (Bacteroidetes) (Figure 3C). At the genus level, there were five genera enriched in the CTL group (*Clostridium, Sutterella, Candidatus_Arthromitus, Bacteroides* and *Parabacteroides*) and six genera in the PZOL group (*Bifidobacterium, Bilophila, Anaeroplasma, Oscillospira, Helicobacter* and *Odoribacter*). (Figure 3D). BioProject accession number PRJNA751763 (www.ncbi.nlm.nih.gov (accessed on 3 August 2021)).

### 2.3. Pantoprazole-Induced Alterations in mRNA and Proteins of the Epithelial Barrier Integrity in Ileum but Not in Colon

**Figure 4:**
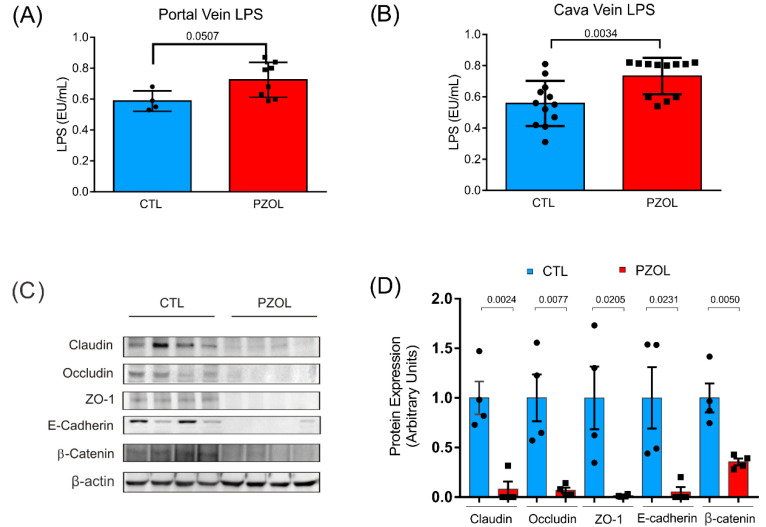
Pantoprazole induced alterations in epithelial barrier integrity in ileum. (**A**) LPS levels in portal vein (CTL: *n* = 4 and PZOL: *n* = 8) and (**B**) cava vein (CTL: *n* = 12 and PZOL: *n* = 12); (**C**) tissue levels of tight junction proteins (claudin, occludin and ZO-1), and adherens junction proteins (e-cadherin and beta-catenin) and (**D**) quantification of the Western blots (CTL: *n* = 4 and PZOL: *n* = 4). Data are expressed as mean ± standard deviation and *t*-test statistical comparisons.

Previous data showed that the modulation of microbiota can induce alterations in epithelial barrier integrity, which in most cases is accompanied by an increase in circulating LPS. Since LPS from microbiota reach the liver through the portal vein before reaching peripheral circulation, we determined LPS levels in the portal veins and peripheral veins of the CTL and PZOL groups. The results showed that LPS was higher in the PZOL group in both the portal (CTL: 0.5875 ± 0.0665 and PZOL: 0.725 ± 0.1128; *p*-value 0.05) and cava (CTL: 0.5575 ± 0.1444 and PZOL: 0.7331 ± 0.1161; *p*-value 0.0034) veins, suggesting that this bacterial lipid translocates the intestinal epithelial barrier (Figure 4A,B). 

**Figure 5:**
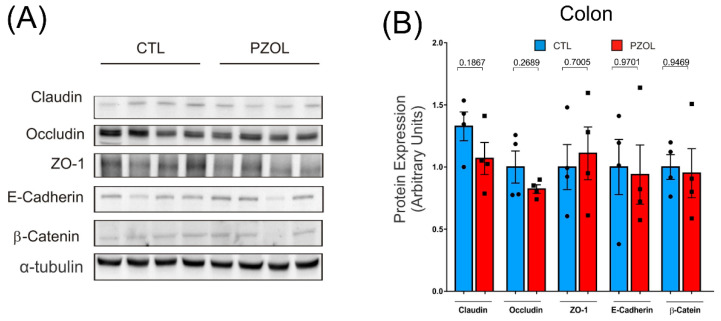
Pantoprazole does not induce alterations in epithelial barrier integrity in colon. (**A**) Tissue levels of tight junction proteins (claudin, occludin and ZO-1), and tissue levels of adherens junction proteins (e-cadherin and beta-catenin); (**B**) quantification of the Western blots (*n* = 4 to 8 animals per group). Data are expressed as mean ± standard deviation and *t*-test statistical comparisons.

We next investigated the effect of pantoprazole on the proteins of the intestinal epithelium barrier in the ileum and colon. The results showed that 60 days of treatment with pantoprazole induced a reduction in tight junction proteins claudin (CTL: 0.9375 ± 0.3377 and PZOL: 0.4712 ± 0.1813 *p*-value 0.0040), occludin (CTL: 0.4325 ± 0.4444 and PZOL: 0.1 ± 0.0886; *p*-value 0.1927) and ZO-1 (CTL: 1 ± 0.2084 and PZOL: 0.001 ± 0.0008; *p* < 0.0001) in the ileum (Figure 4C,D). Moreover, the adherens junctions E-cadherin (CTL: 1 ± 0.5660 and PZOL: 0.0582 ± 0.1145; *p*-value 0.0286) and beta-catenin (CTL: 1.01 ±0.2940 and PZOL: 0.4325 ± 0.0386; *p*-value 0.0080) were also decreased in ileum (Figure 4C,D). Analyzing these proteins in the colon, we observed similar levels between the groups claudin (CTL: 1.3225 ± 0.2315 and PZOL: 1.0675 ± 0.2519 *p*-value 0.1867), occludin (CTL: 1.02 ± 0.2284 and PZOL: 0.87 ± 0.0920; *p*-value 0.2689) and ZO-1 (CTL: 1.0125 ± 0.3531 and PZOL: 1.125 ± 0.4313; *p*-value 0.7005), E-cadherin (CTL: 0.975 ± 0.4260 and PZOL: 0.9875 ± 0.4761; *p*-value 0.9701) and beta-catenin (CTL: 0.9925 ± 0.1635 and PZOL: 0.9775 ± 0.3995; *p*-value 0.9469) (Figure 5A,B).

**Figure 6:**
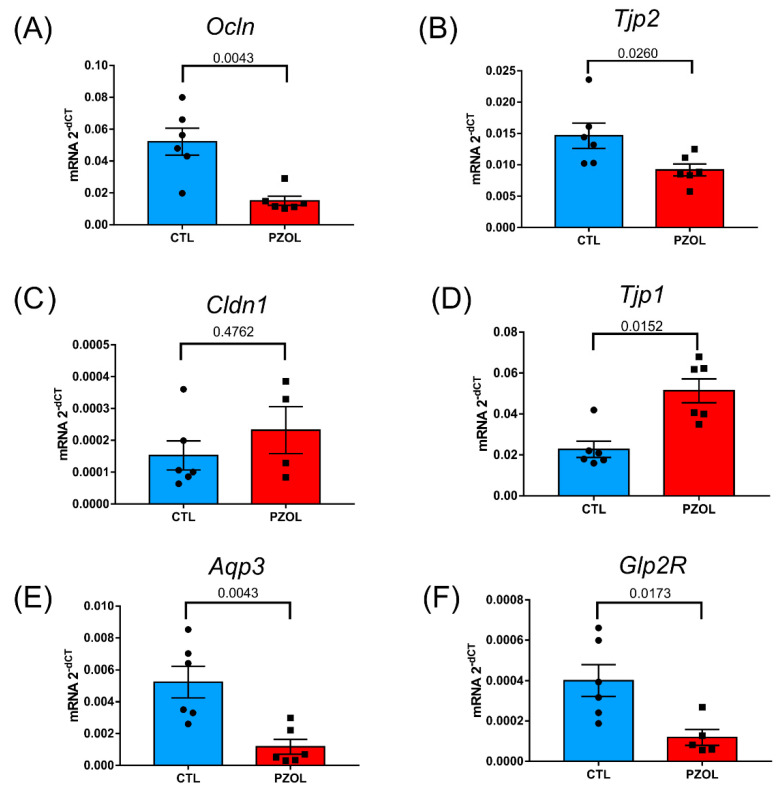
Pantoprazole induced alterations in mRNA of proteins from the epithelial barrier in ileum. mRNA levels of tight junction proteins (**A**) *occludin* (CTL: *n* = 6 and PZOL: *n* = 6), (**B**) *ZO-2* (CTL: *n* = 4 and PZOL: *n* = 4), (**C**) *claudin* (CTL: *n* = 6 and PZOL: *n* = 4), (**D**) *ZO-1* (CTL: *n* = 6 and PZOL: *n *= 6), (**E**) *aquaporin 3* (CTL: *n* = 6 and PZOL: *n* = 6), (**F**) *GLP2R* (CTL: *n* = 6 and PZOL: *n* = 5). Mean ± standard deviation and *t*-test statistical comparisons.

The reduction in protein levels may be a consequence of reduced protein synthesis and/or increased protein degradation. We then investigated the expression of mRNA of the proteins of the intestinal epithelial barrier in the ileum. The results showed that there was a decrease in *OCLN* mRNA (CTL: 0.05216 ± 0.002065 and PZOL: 0.0150 ± 0.0070; *p*-value 0.0043; Figure 6A) and *TJP2* mRNA (CTL: 0.0146 ± 0.0020 and PZOL: 0.0091 ± 0.0023; *p*-value 0.0260; Figure 6B), but no change in *CLDN1* mRNA (CTL: 0.0001524 ± 0.0001118 and PZOL: 0.0002318 ± 0.0001475; *p*-value 0.4762; Figure 6C) and an increase in *TJP1* mRNA (CTL: 0.02272 ± 0.0096 and PZOL: 0.0512 ± 0.01425; *p*-value 0.0152; Figure 6D). In addition, there was a decrease in *Aqp3* mRNA expression (CTL: 0.0052 ± 0.0024 and PZOL: 0.0011 ± 0.0011; *p*-value 0.0043; Figure 6E) and *GLP2R* mRNA (CTL: 0.0004 ± 0.0001 and PZOL: 0.0001 ± 3.953 × 10^−5^; *p*-value 0.0173; Figure 6F). 

**Figure 7:**
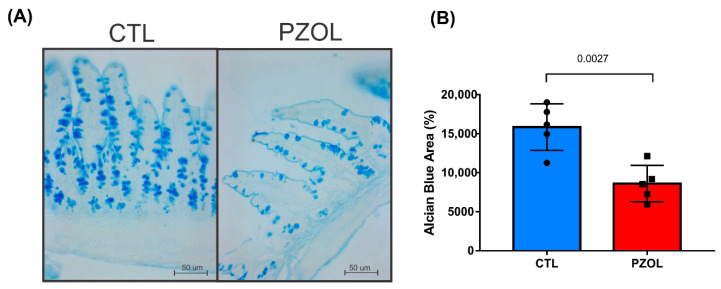
Pantoprazole induced loss of intestinal barrier functions. (**A**) Representative alcian blue (pH 2.5) stained section in the ileum: CTL and PZOL groups; (**B**) AB quantification graph of CTL and PZOL groups expressed as mean ± standard deviation *t*-test statistical comparisons. Images obtained with 20× objective. (*n* = 5 animals per group).

Alcian blue (AB) staining was used to visualize the total (neutral and acid) mucins in the ileum. A lower protein level of mucin was found in the ileum in the PZOL group compared to the CTL group, with the differences being statistically significant (CTL: 15,835 ± 2984 and PZOL: 8609 ± 2327; *p*-value 0.0027) (Figure 7). Our data suggest that animals treated with pantoprazole for 60 days had altered intestinal permeability. 

In preliminary experiments, we investigated the effect of 30 days of pantoprazole on microbiota modulation and on intestinal permeability (Supplemental Figure S1A–C). The results showed that 30 days of pantoprazole did not change microbiota composition nor change proteins’ levels of mucin (Supplemental Figure S1D,E).

### 2.4. Pantoprazole-Induced Liver Fibrosis and Inflammation 

**Figure 8:**
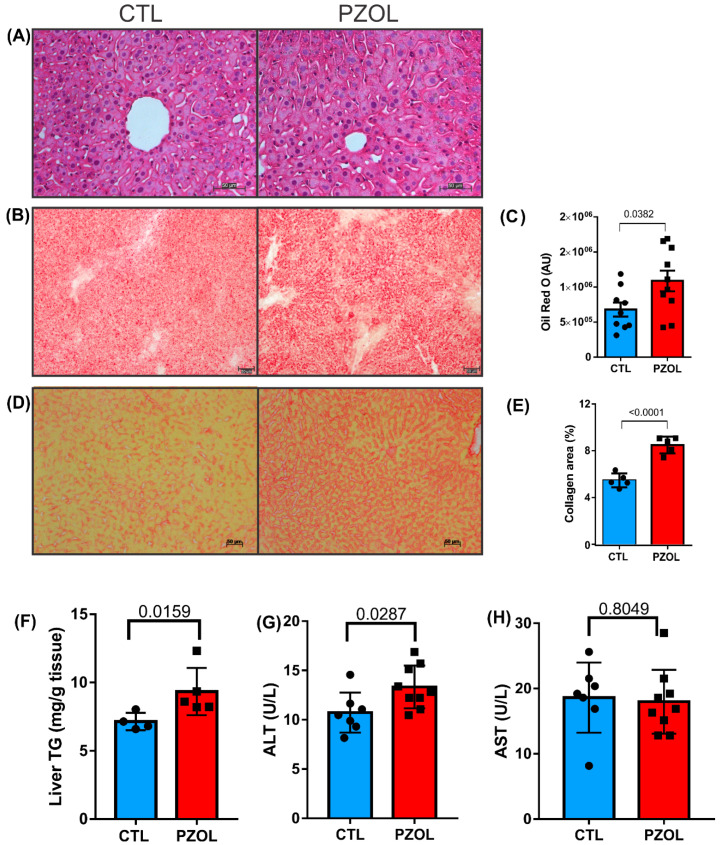
Pantoprazole induced liver inflammation and fibrosis. (**A**) Representative HE images: CTL and PZOL groups; (**B**) representative ORO images: CTL and PZOL groups; (**C**) ORO quantification graph of CTL (*n* = 9) and PZOL (*n* = 10); (**D**) representative PS images; (**E**) PS quantification graph of CTL (*n* = 5) and PZOL (*n* = 5) groups. Images obtained with 20× objective. (**F**) Liver TG quantification graph of CTL (*n* = 4) and PZOL (*n* = 4) groups. (**G**,**H**) Serum ALT and AST quantification graph of CTL (*n* = 7) and PZOL groups (*n* = 9). Mean ± standard deviation and *t*-test statistical comparisons.

Next, we analyzed the effect of pantoprazole treatment in the liver of mice. The morphological analysis of the liver tissue via hematoxylin and eosin staining (Figure 8A) suggests that animals treated with pantoprazole for 60 days might have had microvesicular steatosis, but it is difficult to confirm with this staining. In order to confirm this finding, the hepatic content of neutral lipids was evaluated through Oil-Red-O (ORO) staining (Figure 8B). Our data revealed that the hepatic content of neutral lipids in the PZOL group was 60% higher than in the CTL group (CTL: 679,600 ± 99,270 and PZOL: 1,088,000 ± 147,100; *p*-value 0.0382; Figure 8C). Furthermore, the analysis of collagen content in liver tissue (Figure 8D) showed that animals treated with pantoprazole for 60 days showed higher collagen labeling than animals in the respective control group (CTL: 5.082 ± 0.3889 and PZOL: 9.417 ± 0.3789; *p*-value < 0.0001; Figure 8E). Liver triglycerides (TG) contents in the PZOL group were increased compared to those in the CTL group (CTL: 7.14 ± 0.6351 and PZOL: 9.336 ± 1.731; *p*-value < 0.0159 Figure 8F). The evaluation of liver enzymes showed elevated serum values of ALT (CTL: 10.73 ± 2.0400 and PZOL: 13.32 ± 2.166; *p*-value < 0.0287 Figure 8G) but not AST (CTL: 18.62 ± 5.376 and PZOL: 17.98 ± 4.892; *p*-value < 0.8049 Figure 8H) in the PZOL group compared with the control group.

**Figure 9:**
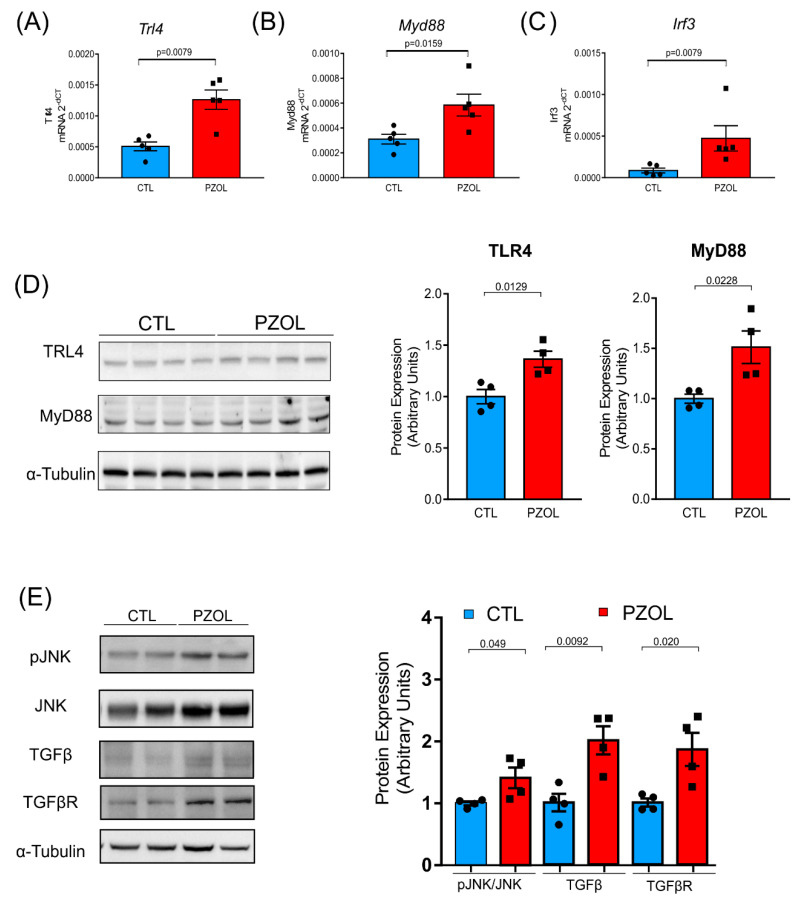
Inflammatory and TGFβ signaling in liver of pantoprazole-treated mice. mRNA levels of (**A**) *TLR4*; (**B**) *MyD88*; (**C**) *IRF3* (*n* = 5 animals per group); (**D**) representative Western blots of TLR4 and MyD88; and quantification of the Western blots (*n* = 4 animals per group); (**E**) representative Western blots of phosphorylated JNK and total TGFβ, TGFβ receptor and of α-tubulin of CTL and PZOL-treated mice and quantification of the Western blots (*n* = 4 animals per group). Mean ± standard deviation and *t*-test statistical comparisons.

The increase in systemic LPS in pantoprazole-treated mice might be responsible for the increase in liver inflammation and/or fibrosis. LPS is the ligand of TLR4, and thus, we decided to analyze the TLR4 signaling and inflammatory pathway. We performed *TLR4*, *MYD88* and *IRF3* gene expression analysis, and the results showed that the PZOL group had a clear increase in the expression of these three genes, compared to the CTL group (CTL: *TLR4*: 0.0005 ± 0.0001 and PZOL: 0.0012 ± 0.0003; *p*-value 0.0079; *MYD88*: CTL: 0.0003 ± 8.785 × 10^−5^ and PZOL: 0.0005 ± 0.0001; *p*-value 0.0152; *IRF3*: CTL: 8.674 × 10^−5^ ± 6.518 × 10^−5^ and PZOL: 0.0004 ± 0.0003; *p*-value 0.0079; Figure 9A–C). Moreover, we also investigated the protein levels of TLR4, MyD88 and downstream signaling JNK phosphorylation. Pantoprazole-treated mice had increased TLR4 (CTL: 1.004 ± 0.1376 and PZOL: 1.34 ± 0.1557; *p*-value 0.0178) and MyD88 (CTL: 1.007 ± 0.046 and PZOL: 1.237 ± 0.162 *p*-value 0.0228) protein expression (Figure 9D) and increased JNK (pJNK/JNK: CTL: 1.017 ± 0.0635 and PZOL: 1.422 ± 0.3303; *p*-value < 0.049) phosphorylation (Figure 9E). Furthermore, we observed an increase in TGFβ (CTL: 1.017 ± 0.2817 and PZOL: 2.122 ± 0.452; *p*-value < 0.0092) and in its receptor (TGFβR: CTL: 0.9889 ± 0.1281 and PZOL: 1.896 ± 0.5311; *p*-value 0.020) in pantoprazole-treated mice (Figure 9E).

## 3. Discussion

The present study demonstrated that chronic treatment with PPIs induces clear changes in intestinal microbiota and the intestinal barrier. Additionally, the impairment on intestinal barrier integrity was associated with an increase in LPS circulating levels, which activates a hepatic signaling cascade in the liver, accompanied by an increase in liver microvesicular steatosis and fibrosis. Our data are in accordance with Takashima et al., who demonstrated that PPIs enhance intestinal permeability, associated with changes in microbiota composition [36]. However, in our study, we went further and investigated the implications of these changes in intestinal permeability and dysbiosis on hepatic injury.

The microbiota composition in the gastric fluid of PPI-treated animals and humans showed an increase in microbial diversity [14,22,37,38]. Our results demonstrated an increase in alpha diversity in the feces of mice after 60 days of PPI treatment. This is an expected result since the main action of PPIs is to reduce gastric acid secretion, inducing an increase in gastric pH. In some studies, microbiota composition in the feces was also investigated, and although not uniformly observed, most studies showed that the chronic use of PPIs modulates microbiota diversity and the abundance of commensals in the colon [14,22,37,38,39]. 

The changes in microbiota induced by pantoprazole were dramatic, starting at the phylum level with an increase in the abundance of Firmicutes and Proteobacteria, a reduction in the Bacteriodetes and significant increase in the Firmicutes/Bacteroidetes ratio, which is a marker of inflammatory processes [23]. The alteration of the Proteobacteria phylum was marked by the increase in bacteria of the *Bilophila* and *Helicobacter* genus. The *Bilophila* genus have already been described in association with the loss of intestinal barrier function, inflammation, alterations in glucose metabolism and hepatic steatosis [39]. *Helicobacter* can indirectly induce insulin resistance (IR) and NAFLD by generating chronic inflammation or directly by activating signaling pathways [40].

*Helicobacter pylori* (*H. pylori*) is strictly dependent on intragastric pH since it enters the replicative phase at pH 6–7, and at pH 3–6, it transforms into its coccoid form, which is resistant to antibiotics [41]. In this way, previous to the use of antibiotics to treat *H. Pylori*, it is recommended to treated patients with PPIs in order to change H pylori form from the resistant to the replication phase, in which it is more susceptible to the action of the antibiotic.

It is important to emphasize that these changes in microbiota were accompanied by an increase in circulating levels of LPS. Firmicutes and Proteobacteria are two main phyla that have LPS in plasma membrane, and these two phyla were increased in PPI-treated mice. 

Although LPS has a role in the development of insulin resistance (IR), this hormonal resistance has more complex mechanisms, which are not limited to modulations of microbiota [29,30,31]. Although pantoprazole induced marked changes in microbiota and in portal and cava LPS levels, we did not observe systemic IR. On the other hand, there was an improvement in insulin sensitivity associated with a reduction in fasting glucose levels. These improvements in insulin sensitivity and in glucose metabolism have also been described in type 2 diabetic patients using PPIs [39]. Although we did not investigate the molecular mechanism for this improvement in insulin sensitivity, previous data showed that the increase in gastrin levels induced by PPIs possibly acts as an incretin mimetic, improving insulin secretion and glucose metabolism [42,43,44].

Previous data showed that under normal conditions, gastric acid acts as a kind of barrier, impairing the progression down to the lower GI tract of bacteria not well-adapted to low pH [37]. Treatment with PPIs removes this barrier, allowing colonization by these bacteria, which can also influence the ecological equilibrium of the microbiota in the lower GI tract. Another mechanism by which PPIs can alter microbiota is through a direct antimicrobial effect of PPIs, acting in ATPases of bacteria, which are very similar to the human H+/K+ ATPase targeted by PPIs [45]. However, since we only observed changes in microbiota after 60 days of PPIs, we can suggest that the changes in pH might have the main role in the modulation of microbiota.

PPIs induce a clear alteration in the epithelial intestinal barrier, characterized by a decrease in tight junction proteins, also associated with a decrease in adherents’ proteins. The decrease in tight junction proteins such as occludin may be a consequence of reduced synthesis. However, ZO-1 protein reduction certainly is independent of mRNA, which was increased, probably trying to compensate the reduction in protein tissue levels. In parallel, there was also a decrease in the mRNA of aquaporin 3 and of the receptor of GLP2, which are also important for the integrity of the intestinal barrier [46,47,48]. 

The alterations in the intestinal barrier have consequences in liver lipid storage and inflammation. Our data showed that the use of PPIs for 60 days can mildly increase the accumulation of TG in liver and also the serum values of ALT. PPI induced microvesicular steatosis, which might be a more severe form of steatosis [49]. In accordance, there was also an increase in markers of liver fibrosis [50].

Previous data showed that dysbiosis in the gut could influence liver fibrosis [50,51,52] through the translocation of bacteria and/or their products across the intestinal barrier. In this regard, the increase in circulating levels of LPS in the portal vein probably contributed to the increased fibrosis. LPS binds to TLR4 in the liver and induces signaling pathways characterized by an increase in IKKβ/NFκB [53] and JNK activation. Our data showing an increase in the phosphorylation of these two serine kinases indicates an increase in LPS signaling in the liver of PPI-treated mice. Previous data showed that the blockade of TLR4 signaling or the use of antibiotics that reduce the microbiota improves experimental liver fibrosis [51]. It is important to mention that TLR4 signaling to the nucleus uses NFκB through IKKβ, AP-1 through JNK and IRF3 directly, and these three pathways were activated in the livers of PPI-treated mice [53].

TLR4 is expressed in different cells in the liver, including hepatocytes, hepatic stellate cells (HSCs), and Kupffer cells. In HSCs, TLR4 can activate a fibrogenic phenotype, producing chemokines and adhesion molecules that recruit Kupffer cells [31,51,52]. The Kupffer cells increase TGFβ production, which will activate fibrogenesis. Our data show that this mechanism is probably operating in the liver of pantoprazole-treated mice because, in addition to the activation of downstream TLR4 signaling, we also observed an increase in TGFβ tissue levels in these mice. 

Different from our data, a previous report by Lu et al. showed that pantoprazole improved liver fibrosis and suppressed hepatic stellate cell activation in mice. In addition, they also showed in cells that PPIs can downregulate hepatic fibrogenic gene expression via YAP (Yes-associated protein). However, it is important to mention that there are clear methodological differences between our data and this study that can explain the discrepancy in the results. First, in their study, Lu et al. used PPIs for fourteen days in mice, which is probably not sufficient to induce changes in intestinal microbiota and/or in intestinal permeability. The model they used of hepatic injury is completely different from ours, and some of their results are obtained in cell culture. In preliminary experiments, we showed that 30 days of PPIs was not able to change microbiota or intestinal permeability. This explains why we used PPI for 60 days. In this regard, we can suggest that although PPIs for short periods or in vitro can have protective effects on hepatic fibrosis, long-term PPI use can induce dysbiosis, change intestinal permeability and induce hepatic steatosis and fibrosis.

Our data showing that the long-term use of PPIs can alter microbiota and intestinal permeability and contribute to induce microvesicular steatosis and fibrosis are important as an initial model for the study of these interactions in vivo. However, it is important that future studies also investigate the effects of long-term PPIs on animal models of heartburn or GERD.

In summary, our data showed that chronic treatment with pantoprazole induced the modulation of microbiota which was not associated with IR. However, the alteration in intestinal permeability, associated with the increase in circulating levels of LPS, TLR4 downstream signaling and TGFβ, may have an important role in the increased microvesicular steatosis and fibrosis. Finally, this model of PPI-induced changes in microbiota might be useful to investigate liver microvesicular steatosis and fibrosis.

## 4. Materials and Methods

### 4.1. Animal Characterization 

All animal handling and experiments were performed following the National Institute of Health guidelines for the use of experimental animals and were approved by the Care of Animals and Ethical Committee for Animal Research of the State University of Campinas (CEUA Protocol 4924-1/2018). To carry out the study, male C57BL/6J mice were provided by the Multidisciplinary Center for Biological Investigation on Laboratory Animal Science—University of Campinas (Campinas,SP, Brazil). Mice were kept in the animal facility with a constant light/dark cycle (12 h/12 h), room temperature (22 °C) and humidity, and they received standard rodent chow (3.39 kcal/g; Nuvilab CR-1, Nuvital Quimtia, (Colombo, PR, Brazil) and water ad libitum. 

After 1 week of acclimation, 8–12-week-old mice were randomly divided into two experimental groups. Control mice (CTL, *n* ≥ 8) were fed with standard rodent chow and had free access to drinking water and supplement with vehicle by gavage three times a week. Pantoprazole-treated mice (PZOL, *n* ≥ 8) were fed with standard rodent chow and free access to water and supplement with a dose of 150 mg/kg of pantoprazole by gavage three times a week, on Mondays, Wednesdays and Fridays.

### 4.2. Glucose and Insulin Tolerance Test

For the GTT, mice were fasted overnight and received an intraperitoneal (IP) injection of glucose (1 g/kg). Blood samples were collected from the tail, and the glucose level was determined using a glucose monitor (Glucometer; Bayer, Tarrytown, NY, USA) immediately before IP injection and after 30, 60, 90 and 120 min. For the ITT, mice fasted for six hours received an IP injection of insulin (1.5 IU/kg). Blood samples were collected from the tail, and glucose was measured with a glucometer immediately before IP injection and after 5, 10, 15, 20, 25 and 30 min. Insulin tolerance was assessed by glucose clearance over the initial minutes of the insulin challenge and through the rate constant for plasma glucose disappearance (Kitt) [54]. 

### 4.3. Serum Dosage of LPS

Serum samples were obtained from the cava vein and portal vein and diluted to 20% (vol./vol.) with endotoxin-free water and then heated at 70 °C for 10 min to inactivate serum proteins. Then, LPS was quantified using a commercial Limulus Amebocyte Assay kit (Cambrex, Walkersville, MD, USA) according to the manufacturer’s protocol, as previously described [55]. 

### 4.4. TG, ALT, and AST Determination

Liver samples were subjected to lipid extraction as previously described [56,57]. Briefly, the samples were homogenized in a solution containing chloroform and methanol using the BeadBlaster 24 microtube homogenizer (Benchmark Scientific, Inc., Sayreville, NJ, USA) and gently shook overnight at 4 °C. After the incubation, the samples were added with 0.6% NaCl solution and centrifuged (4000× *g* for 20 min at 4 °C) for removal of the organic layer. The organic fraction was dried at room temperature, reconstituted in 100 µL of isopropanol and used for triglycerides’ (TGs’) quantification with an enzymatic/colorimetric kit (LABORLAB, Sao Paulo, SP, Brazil). Serum alanine aminotransferase (ALT) and aspartate aminotransferase (AST) were measured with the kinetic UV method using the ALT (GPT) or AST (GOT) Liquid Stable Reagent according to the manufacturer’s specifications (LABORLAB, Sao Paulo, SP, Brazil). 

### 4.5. Tissue Extraction for Immunoblotting

Mice were fasted for 6 h before procedures. Mice were anesthetized with an intraperitoneal administration of ketamine (100 mg/kg) and xylazine hydrochloride (10 mg/kg), and after the loss of foot, tail and corneal reflexes, they were utilized. Liver and intestine fragments (~100 mg) were collected and homogenized in extraction buffer (10 mmol/L EDTA, 100 mmol/L Tris (pH 7.4), containing 100 mmol/L sodium pyrophosphate, 100 mmol/L sodium fluoride, 10 mmol/L sodium vanadate, 2 mmol/L PMSF and 0.1 mg of aprotinin/mL, 1% Triton-X 100). Samples were centrifuged at 11,000 rpm and 4 °C, and the supernatants were used. The samples were subjected to electrophoresis and Western blotting. The primary antibodies used were anti-phospho-JNK (sc-6254 Santa Cruz), anti-phospho-IkBα (sc-7977 Santa Cruz), anti-JNK (sc-1648 Santa Cruz), anti-TGFβ (ab92486 Abcam), anti-TGFβ Receptor (ab31013 Abcam), anti-TLR4 (ab22048 Abcam), anti-Myd88 (sc-11356 Santa Cruz), anti-ZO-1 (61-7300 Thermo Fisher), anti-Occludin (71-1500 Thermo Fisher), anti-Claudin 1 (#13995 Cell Signalling), anti-E-cadherin (sc-7870 Santa Cruz), anti-Beta catenin (sc-1496 Santa Cruz), anti-α-tubulin (#2144 Cell Signalling) and anti-β-actin (#4967 Cell Signalling) at 1:1000 dilution. The secondary antibody (Thermo Scientific) linked to a peroxidase molecule, at 1:10,000 dilution, reacted with the chemiluminescence solution (ClarityTM Western ECL substrate kit, BioRad ©), and the membranes were developed in photodocumentation (Gel Doc ™ XR, BioRad ©), generating digital files. Later, images were analyzed using the ImageLab software (v. 5.2.1 build 11, BioRad © Laboratories). 

### 4.6. Histology and Morphometric Analysis

Liver fragments were fixed at room temperature by immersion in 4% formaldehyde in 0.1 M phosphate buffer, pH 7.4, dehydrated, bleached and embedded in paraffin [58]. Five micrometer sections were stained with hematoxylin and eosin to analyze the liver morphology or Picro Sirus red (PS) solution to assess the presence or absence of liver fibrosis through the quantification of the interstitial collagen content. The interstitial collagen area was determined for the entire PS-stained liver section by using digitized images captured using the Axio Scope A1 microscope with an Axio Cam MRc digital camera (Carls Zeiss, Oberkochen, Germany) and the Axio Vision Release 4.8.2 software. Colored images were converted to 8 bit, and a default B&W was set. Since we assumed a constant background signal (threshold) for all images, the percentage of positive area for the PS staining of each field was recorded. On average, 15 fields were analyzed for animals under a 20× objective. 

Liver sections for neutral lipid staining with ORO were obtained and processed based on previous studies [59,60]. Briefly, liver fragments were embedded in Tissue-Tek O.C.T. compound and immediately frozen in n-hexane with liquid nitrogen (N_2_). Fifteen micrometer serial cryostat sections were mounted onto aminopropyltriethoxysilane-coated glass slides. Three sections from different parts of the samples (200 μm apart) were disposed per slide, and two slides per animal were analyzed. Liver sections were incubated with ORO for 10 min at room temperature and then rinsed with tap water for 30 min. After rinsing, a water-soluble mounting medium was used prior to observation via microscopy. Four different fields of each section were acquired with an Olympus BX51TF microscope equipped with a digital camera (DP72, Olympus, Tokyo, Japan) under a 20× objective. Colored images were converted to 8 bit. Thereafter, a default red was set, and the threshold was adjusted to evaluate the optical density of the positive area for ORO staining of each field. An individual background signal was assumed for each image. All histological analyses were performed using the ImageJ software (version 1.53e).

The ileum was collected from animals, and this intestinal segment was cleaned and longitudinally opened with the luminal side facing upward. Gently and slowly, the ileum was wrapped around the toothpick to form a Swiss roll. Once the entire length of the ileum was rolled, we placed it into a tissue cassette and fixed it at room temperature by immersion in 4% formaldehyde in 0.1 M phosphate buffer, pH 7.4, dehydrated, bleached and embedded in paraffin [61]. In each cassette, we had an ileum Swiss roll from three different mice. Five micrometer sections were stained with alcian blue pH2.5 (AB) to stain the mucus. Digitalized images were captured using the Axio Scope A1 microscope with an Axio Cam MRc digital camera (Carls Zeiss, Oberkochen, Germany) and the Axio Vision Release 4.8.2 software. Colored images were converted to 8 bit, and a default B&W was set. The percent of mucus-stain-positive area per microvilli area was measured. All histological analyses were performed using the ImageJ software (version 1.53e).

### 4.7. Microbiota Analysis

Fecal samples were collected directly from the rectal ampulla after (*n* = 8) the treatment period and were stored at −80 °C until the analysis. Samples were collected, stored and processed in a controlled environment to minimize the risk of contamination. The genomic DNA from 200 mg of stool was extracted using the QIAamp DNA Stool Mini Kit (Qiagen, Hilden, Germany). A negative control (water from QIAamp DNA Stool Mini Kit) was used from an extraction step to the final sequencing, and a mock microbial DNA community standard was used as a positive control (ZymoBIOMICS Irvine, CA, USA). For each sample, the V3–V4 hyper-variable region of the bacterial 16S rRNA gene was amplified followed by Illumina 16S Metagenomic Sequencing Library Preparation guide [62]. The taxonomic composition of the bacterial communities was obtained by analyzing the V3–V4 region of the 16S rRNA gene using the Illumina^®^ MiSeq platform. The constructions of the DNA sequencing libraries were performed according to the manufacturer’s instructions (Illumina, San Diego, CA, USA) [62] and followed the same flow described by Caporaso et al. (2012) [63]. The fastq sequences were analyzed using the Illumina 16S Metagenomics software which performs the taxonomic classification of the V3–V4 region of the 16S rRNA gene using the DADA2 database (accessed on June 2020) [64]. Paired abundance analyses were performed using the IBM SPSS^®^ 20.0 software (Wilcoxon Signed Ranks Test). The analysis of alpha and beta diversity was performed using the MicrobiomeAnalyst [65,66]. The graphs were generated using GraphPad Prism 7.0. 

### 4.8. qPCR

Total RNA was obtained from the ileum, colon, and liver from both groups of mice using RNeasy Mini Kit from Qiagen as described in the manufacturer’s protocol ( Qiagen Inc, CA, USA). For tissue samples, the first-strand cDNA was synthesized using High Capacity cDNA Reverse Transcription Kit as described in the manufacturer’s protocol (Applied Biosystem, CA, USA). TaqMan and Quant Studio 6 Flex Real-Time PCR System and Data Assist™ software Applied Biosystems, CA, USA) were used to get the relative expression levels of the genes: *Ocln* Mm00500912_m1; *Tjp2* Mm00495620_m1; *Cldn1* Mm00516701_m1; *Tjp1* Mm00493699_m1; *Aqp3* Mm01208560_m1; *Glpr2* Mm01329475_m1. SybrGreen PCR Master Mix (Applied Biosystem, CA, USA) was used to get the relative expression levels of the genes: 150 nM of *Tlr4* (Fw: 5′- GGCCATTGCTGCCAACAT –3′; Rv: 5′- CAACAATCACCTTTCGGCTTTT -3′), 150 nM of *MyD88* forward and reverse primers (Fw: 5′- TCGATGCCTTCATCTGCTATTG -3′; Rv: 5′- GGT CGGATCATCTCCTGCACAAA -3′) and 150 nM of *Irf3* (Fw: 5′- TTCCCGGGAGGGATAAGC -3′; Rv: 5′- GGGCAGAGCGGAAATTCC -3′) [53]. *Gapdh* and *B2m* expression were used as endogenous control, and samples from control mice were used as calibrators. A negative “No Template Control” was also included for each primer pair.

### 4.9. Statistical Analyses

The data were expressed as means ± standard deviation. For statistical analysis, the groups were compared using Student’s *t*-test. The level of significance was set at *p* < 0.05. 

## Data Availability

BioProject accession number PRJNA751763 (www.ncbi.nlm.nih.gov (accessed on 3 August 2021)).

## Suplementary Materials

The following supporting information can be download at: https://www.mdpi.com/article/10.3390/ijms232213766/s1.
