# Supplementary figures and images for: Proton Pump Inhibitor Pantoprazole Modulates Intestinal Microbiota and Induces TLR4 Signaling and Fibrosis in Mouse Liver
Source: Int J Mol Sci. 2022 Nov 9;23(22):13766. doi: 10.3390/ijms232213766 (PMC9693486; doi:10.3390/ijms232213766)

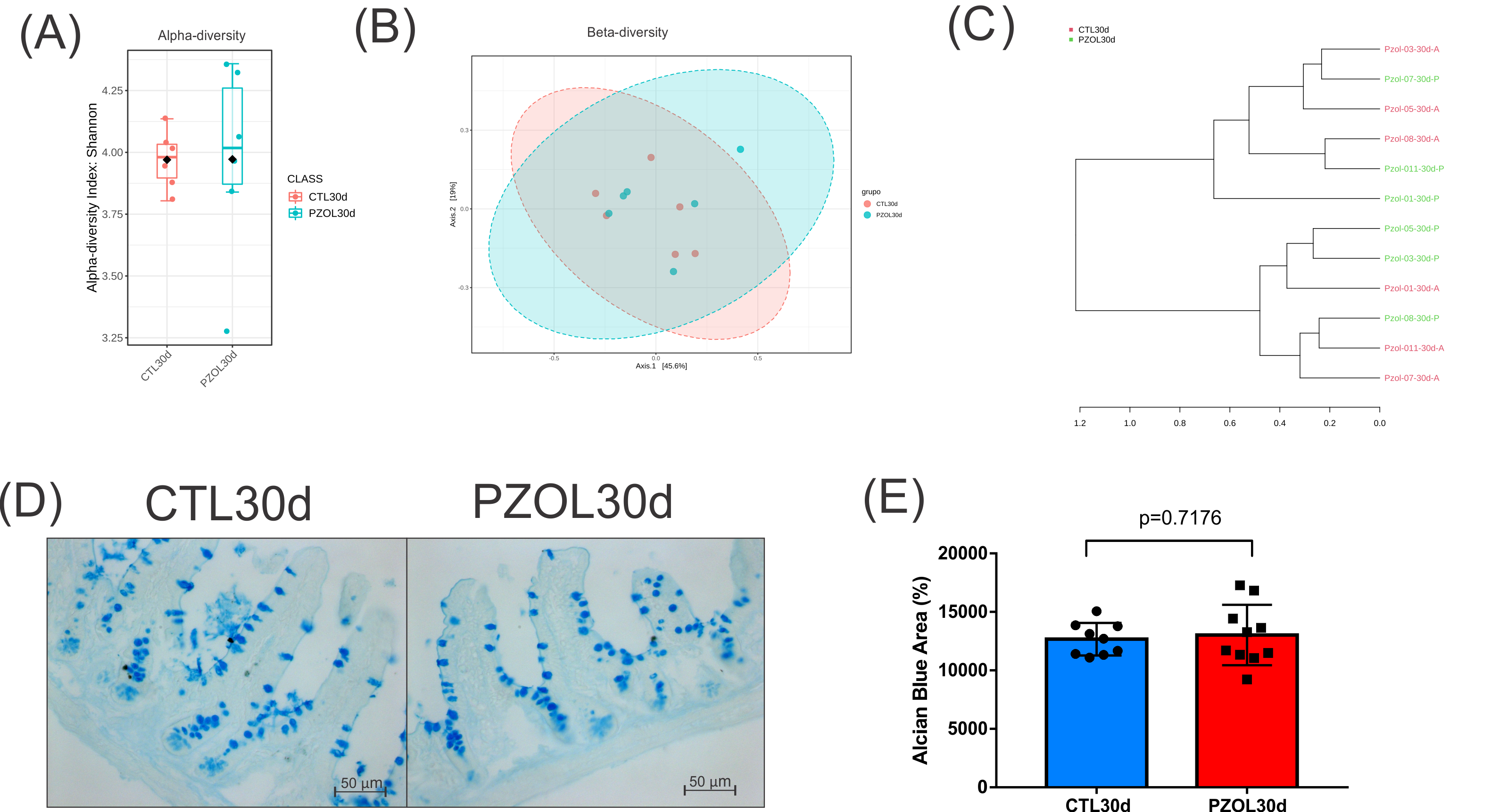

Supplement: Supplementary file 1 [file ijms-23-13766-s001.zip › ijms-1919687-supplementary.tif]
